# Public Grants to Schools for Mothers: The Board of Education's Proposals

**Published:** 1914-09-26

**Authors:** 


### PUBLIC GRANTS TO SCHOOLS FOR MOTHERS.

### The Board of Education's Proposals.

An important step in administrative progress, and in the recognition by the State of its vital interest in the good health and proper rearing of the coming generation of its citizens, has been taken lately by the Board of Education. In regulations which have been presented to both Houses of Parliament, printed, and published, the Board lays down the conditions upon which it will allot public moneys in support of duly-constituted and efficient schools for mothers.

These conditions, as is natural, bear a strong family resemblance to those which the Board laid down only a few weeks ago when dealing with the feeding of school-children (see The Hospital, July 4, 1914, p. 369). Where, in the Board's opinion, the provision made by an institution is adequate and its working is efficient, a grant may be made at the rate of one-half of the approved expenditure. In other cases the Board may either pay at a lower rate or may withhold the grant. In fixing the rate of grant the Board will have regard to the provision made for co-ordinating the work of the institution with that of similar institutions in the same district, of baby clinics and infant dispensaries, and of the school medical service and the sanitary authority; and for keeping records of attendances at the institution and of visits paid to the homes. It is further provided that the institution must be conducted by a responsible body of managers, and a person must be appointed to act as correspondent on behalf of the managers; and also that the institution must not be conducted for private profit or farmed out to any member of the staff.

### The Views of the Board.

Accompanying these formal regulations is a Memorandum (Circular 852) containing an explicit summary of the views of the Board of Education upon the whole subject of schools for mothers. The medical inspection of school-children has brought home to the Board what the medical profession has been preaching for years—namely, that a vast amount of ill-health and defective development exists among the children of the labouring classes which is attributable mainly to insufficient knowledge of the elementary rules of health among the mothers. The Board also professes the conviction that much of this suffering and ill-health can be prevented by the establishment of schools for mothers acting in co-operation with local education and sanitary authorities. Such schools for mothers are defined as primarily educational institutions providing training and instruction for mothers in the care and management of infants and little children. The provision of specific medical and surgical advice and treatment should only be incidental, thus distinguishing a school from a baby clinic or an infant dispensary.

The Board proceeds to indicate certain considerations which should be borne in mind in the organisation of a school for mothers. Systematic instruction and training should form a definite part of the activities of the institution, and facilities should be offered for attendance at classes in subjects bearing directly on the work undertaken, such as care and management of infants and young children, domestic and personal hygiene, home nursing, and cookery. The instruction should be of a simple character, and as far as possible practical work should be included.

### The Limits of Medical Supervision.

A syllabus of instruction is to be submitted to the Board, and the right is reserved of requiring alterations to be made in it. The Board will not insist on the possession of any specified qualifications by teachers, provided they are competent to give efficient instruction in the subject of the course. This cryptic utterance may perhaps be taken to mean that the Board will not inquire into the quality of the instruction given, unless this should be so extraordinarily bad as to lead to open scandal.

In respect of infant consultations no such laxity of control will prevail. It is definitely required that these should be held as far as possible under medical supervision, and that there should be a medical officer on the staff of the institution at the head of, and responsible for, this department of the work. There should also be a superintendent at each institution, acting under the direction of the medical officer; and this superintendent should be a qualified nurse, and should possess definite qualifications for her work. Consultations for infants should be held not less often than once a fortnight, and whenever practicable they should take place once a week or even oftener. The Memorandum also gives much useful information upon such points as home visiting, the disposition of institutions so as to avoid overlapping and competition, the questions of co-ordination with the school medical service, the local education authority, and the sanitary authority. The advantages of close touch with the health visitors who work under the medical officers of health are also emphasised, especially in London. Careful record-keeping is inculcated, and a schedule is annexed to the Memorandum showing what information is required of those who apply for grants, and how it should be analysed and displayed.

